# Piezo1 mediates stretch-induced Wnt/β-catenin signaling and extracellular matrix remodeling in aging asthmatic airway smooth muscle

**DOI:** 10.1093/ajrcmb/aanag058

**Published:** 2026-03-25

**Authors:** Niyati A Borkar, Daniel A Pfeffer-Kleemann, Samantha K Hamrick, Maunick L Koloko Ngassie, Michael A Thompson, Christina M Pabelick, Y S Prakash

**Affiliations:** Department of Anesthesiology and Perioperative Medicine, Mayo Clinic, Rochester, MN, United States; Department of Anesthesiology and Perioperative Medicine, Mayo Clinic, Rochester, MN, United States; Department of Anesthesiology and Perioperative Medicine, Mayo Clinic, Rochester, MN, United States; Department of Anesthesiology and Perioperative Medicine, Mayo Clinic, Rochester, MN, United States; Department of Anesthesiology and Perioperative Medicine, Mayo Clinic, Rochester, MN, United States; Department of Anesthesiology and Perioperative Medicine, Mayo Clinic, Rochester, MN, United States; Department of Physiology and Biomedical Engineering, Mayo Clinic, Rochester, MN, United States; Department of Anesthesiology and Perioperative Medicine, Mayo Clinic, Rochester, MN, United States; Department of Physiology and Biomedical Engineering, Mayo Clinic, Rochester, MN, United States

**Keywords:** mechanobiology, asthma in the elderly, airway smooth muscle, remodeling, Wnt signaling

## Abstract

Asthma in the elderly (AIE) represents a unique challenge, necessitating a better understanding of the mechanisms that drive airway hyperreactivity and remodeling (thickened, fibrotic airways). Normal aging is associated with increased lung stiffness, and thus the influence of mechanical forces on bronchial airways (breathing, sighs, CPAP) likely changes, potentially increasing with AIE and resultant remodeling, overall highlighting the need to understand the importance of mechanobiology in aging and AIE. Here, mechanosensitive Piezo (Pz) channels are highly expressed in the lung, including within airway epithelium and smooth muscle (ASM), but their specific roles, particularly with aging, remain largely unexplored. In this study, we used human ASM cells from young vs old (≥65 y) non-asthmatics vs asthmatics to test the hypothesis that normal aging blunts Pz expression and functionality at baseline as well as in response to mechanical stretch, whereas asthma, particularly, AIE ASM leads to enhanced Pz expression promoting fibrosis and remodeling. Both Pz1 and Pz2 were expressed in ASM and increased in AIE. Oscillatory mechanical stretch mimicking breathing with superimposed static stretch increased Pz, especially in AIE: effects exacerbated by Pz activator Yoda1 and blunted by Pz inhibitor GsMTx4. Stretch or Yoda1 increased Wnt3a, β-catenin, and GSK3β activation, particularly in AIE, while ASM proliferation and extracellular matrix production remained preserved or even heightened in elderly ASM. Conversely, Pz1 inhibition by GsMTx4 potently reduced extracellular matrix (ECM) deposition, particularly in AIE cells, highlighting Pz1’s pivotal role in modulating ECM deposition. Overall, our findings indicate Pz1 as a mechanosensitive regulator of airway remodeling in AIE

Clinical Relevance These findings highlight Piezo1-mediated mechanotransduction as a novel and clinically relevant pathway linking airway stretch to Wnt/β-catenin–driven extracellular matrix remodeling. This pathway may contribute to therapeutically resistant and age-related airway diseases such as asthma.

## Introduction

With a global increase in the elderly population (>65 years of age), it is all the more important to understand not only structural and functional changes in the airways in the context of healthy aging but also in diseases such as asthma.^1^^,^^2^ Asthma across the age spectrum involves chronic inflammation associated with airway hyperreactivity and largely irreversible airway remodeling.^3-5^ In this regard, clinical studies have shown that while asthma incidence and prevalence are similar across the adult age spectrum, asthma in the elderly (AIE) is more severe, has greater morbidity and mortality, and may be more resistant to standard therapies.^6-8^ Understanding AIE and its underlying mechanisms is confounded by normal aging-related decrease in lung function, senile emphysema, and thicker and more fibrotic bronchial airways.^9^^,^^10^ Asthma affects over 300 million individuals, including >20 million in the U.S. alone, thereby representing a major healthcare burden and further necessitating an improved understanding of its pathophysiology and novel therapies to alleviate symptoms.^11^ One impact of increased fibrosis with aging is greater stiffness and the mechanical environment within which cells of the airway function in normal aging or particularly in AIE. AIE is also characterized by such structural and functional changes in the airways, but the contribution of different cell types or underlying mechanisms remains less clear.^12^ In addition to their well-known role in airway contractility and hyperresponsiveness, airway smooth muscle (ASM) cells contribute to remodeling via increased cell proliferation and hypertrophy, and production of extracellular matrix (ECM) proteins that contribute to fibrosis.^5^^,^^13^ We previously showed that ASM shows increased ECM production with normal aging, including some components such as collagens that contribute to airway stiffness in asthma.^14^^,^^15^ However, the mechanisms that contribute to age-related ASM ECM, or changes in ASM of individuals with AIE, are not known.

Given the potential importance of mechanical forces in airways with aging and/or AIE, mechanosensitive pathways would be important to explore in the context of airway remodeling. There has been considerable interest in the role of mechanosensitive Piezo (Pz) channels, Piezo 1 (Pz1), and Piezo 2 (Pz2) that translate mechanical forces into biochemical signals in a multitude of organ systems and cell types. Pz channels are large nonselective cationic channels activated by membrane tension and modulate critical processes such as proliferation, migration, differentiation, and ECM production.^16^^,^^17^ While Pz1 is ubiquitously expressed, Pz2 is thought to be more selectively found in neuronal subtypes.^18^^,^^19^ However, Pz channels are highly expressed in the lung and have been implicated in lung development, vascular function, and epithelial integrity.^20^^,^^21^ We previously showed that both developing and adult ASM cells express Pz channels (particularly Pz1), and contribute to stretch-activated [Ca^2+^]_*i*_ responses,^20^ interacting with microdomain pathways such as STIM1/Orai1 and contributing to Ca^2+^ regulation in adult ASM.^22^^,^^23^ However, these represent acute responses of Pz channels in developing or adult airways. In contrast, chronic effects of Pz channels in airways (eg, toward remodeling) are unknown. We have demonstrated a role for Pz1 in mechanical stretch-induced fibroblast remodeling and ECM formation via the ERK pathway in human lung fibroblasts.^24^ However, the expression and functional contribution of Pz channels in aging airways, including in the context of AIE, are unknown. Furthermore, upstream factors that drive Pz channel expression or modulate functionality have not been well-explored. We previously showed that in developing ASM, stretch itself can modulate Pz expression.^20^ In other cell types, inflammation has been shown to increase Pz1 expression.^25^ Whether these upstream factors play a role in regulating Pz expression or function in aging or AIE airways remains unknown.

Beyond Ca^2+^, the mechanisms by which Pz channels have functional effects in cells are still under investigation.^26^ Pz channels have been shown to interact with pathways such as RhoA/ROCK, Wnt, and β-catenin that also happen to be responsive to stretch and are involved in airway remodeling.^27-29^ Although the Wnt subtypes involved in airways are still under investigation, in vitro studies in adult human ASM show increased Wnt5a in mild to moderate asthmatics compared to healthy individuals, while increased actin filaments and cellular contraction occur with exogenous Wnt5a.^30^^,^^31^ Mouse models of asthma show changes in Wnt signaling resulting in increased airway wall inflammation and remodeling.^32^ Whether and how Pz channels (Pz1 and Pz2) interact with Wnt and β-catenin in ASM in the context of aging or AIE is unknown.

In the present study, we used human ASM cells from young vs elderly (>65 y) non-asthmatics vs asthmatics to test the hypothesis that normal aging blunts Pz expression and functionality at baseline, as well as when exposed to mechanical stretch, when compared to other ASM groups, whereas AIE leads to enhanced Pz that promotes fibrosis and remodeling. We observe that the magnitude of stretch-induced upregulation of Pz expression is comparatively lower in non-asthmatic elderly ASM compared to young and asthmatic counterparts, whereas asthmatic ASM cells maintain a heightened Pz expression regardless of age. We further propose that despite age-related changes in expression, downstream mechanosensitive responses (eg, proliferation and ECM deposition) may remain preserved or even heightened under stretch or pharmacologically activated conditions. We focused on exploring baseline and stretch-induced changes in Pz channel expression across these groups, and the downstream impact of altered Pz expression or function on ASM cell proliferation, ECM production, and signaling pathways such as Wnt and β-catenin.

## Materials and methods

### Human ASM cells

As described previously,^33^^,^^34^ all protocols involving human lung tissue and primary human ASM cells were approved by the Mayo Clinic Institutional Review Board (#08-002518 and #16-009655). Additional details are available in the Supplementary Material (Table S1).

### Chemicals, drugs, inhibitors, and antibodies

Cell culture reagents, DMEM/F12, antibiotic-antimycotic (AbAm), and 0.25% trypsin-EDTA were obtained from Invitrogen (MA, USA). Charcoal-stripped FBS was procured from Sigma-Aldrich (MA, USA). Protease and phosphatase inhibitor cocktail (P178445) was purchased from Thermo Fisher Scientific (MA, USA). Yoda1 (Pz1 agonist; 5586) was purchased from Tocris (Bristol, U.K.). GsMTx4 (Pz inhibitor; HY-P1410) was purchased from MedChemExpress (NJ, USA). Control shRNA lentiviral particles-A (sc-108080) and Pz1 shRNA lentiviral particles (sc-93227-V) were procured from Santa Cruz Biotechnology (TX, USA). Primary antibodies are detailed in the Supplementary Material. Other chemicals and reagents were procured from Sigma-Aldrich unless otherwise indicated.

### Immunofluorescence

Standard immunofluorescence was performed as detailed in the Supplementary Material.

### Cell stretch

Human ASM cells were seeded at 20 000 cells per well onto type I collagen-coated Flexcell biaxial 6-well plates (BioFlex-I; Flexcell International Corp., NC, USA). Once cells reached 60%-70% confluency, they were switched to serum-free medium and then subjected to a 24-hour stretch regimen using a Flexcell FX-4000 Tension System (37 °C, 5% CO_2_, humidified incubator). This regimen involved a sinusoidal 5% cyclic strain at 15 cycles per minute (mimicking normal breathing) either alone (control) or combined with a continuous 10% static stretch (simulating airway stretch from treatments like continuous positive airway pressure; CPAP). Any interventions, such as shRNA transduction, were carried out on the cells in regular plates, and then seeded in the Flexcell plates before the stretch exposure began.

### Cell proliferation

*Bright-field cell counting:* As outlined in the Supplementary Material.

*CyQuant proliferation assay:* Proliferation of ASM cells, cultured in 96-well plates, was assessed using the CyQuant NF kit (Invitrogen, MA, USA) as detailed in the Supplementary Material.

### ECM deposition

Human ASM cells were seeded in 96-well black-walled, clear-bottom plates and allowed to adhere overnight, followed by a 48-hour period of serum starvation. Subsequently, cells were treated for 24 hours in fresh serum-free media containing Yoda1 or GsMTx4. After treatment, the ECM deposition of proteins was calculated as detailed in the Supplementary Material.

### Protein analyses

ASM cell lysates were prepared using cell lysis buffer from Cell Signaling Technologies (MA, USA) supplemented with protease and phosphatase inhibitors, following established protocols.^34^^,^^35^ The protein concentration of the resulting supernatants was then determined using the DC protein Assay kit (BioRad, CA, USA) and assayed as detailed in the Supplementary Material.

### shRNA lentiviral particle transduction

The detailed procedure is as outlined in the Supplementary Material.

### Statistics

All experiments were performed on ASM cells from at least 5 patients and performed in duplicate for each patient sample. Statistical comparisons were made using either Student’s *t*-test or two-way ANOVA as appropriate, followed by Tukey’s post hoc multiple comparisons test using GraphPad Prism, Version 10.2.0 for Windows (GraphPad Software; www.graphpad.com). Statistical significance was tested at a level of *P* < .05. All values are expressed as Mean ± SEM (minimum to maximum, showing all points).

## Results

### Pz1 and Pz2 localization in human ASM cells

Immunohistochemical analysis of human lung tissue showed that both Pz1 and Pz2 are present in the airways, specifically within the ASM layer, as confirmed by co-staining with α-SMA (Figure 1A). Immunostaining of isolated human ASM cells revealed distinct subcellular localization: Pz1 was predominantly found near the cell membrane, whereas Pz2 was surprisingly intracellular (Figure 1B and C).

**Figure 1:**
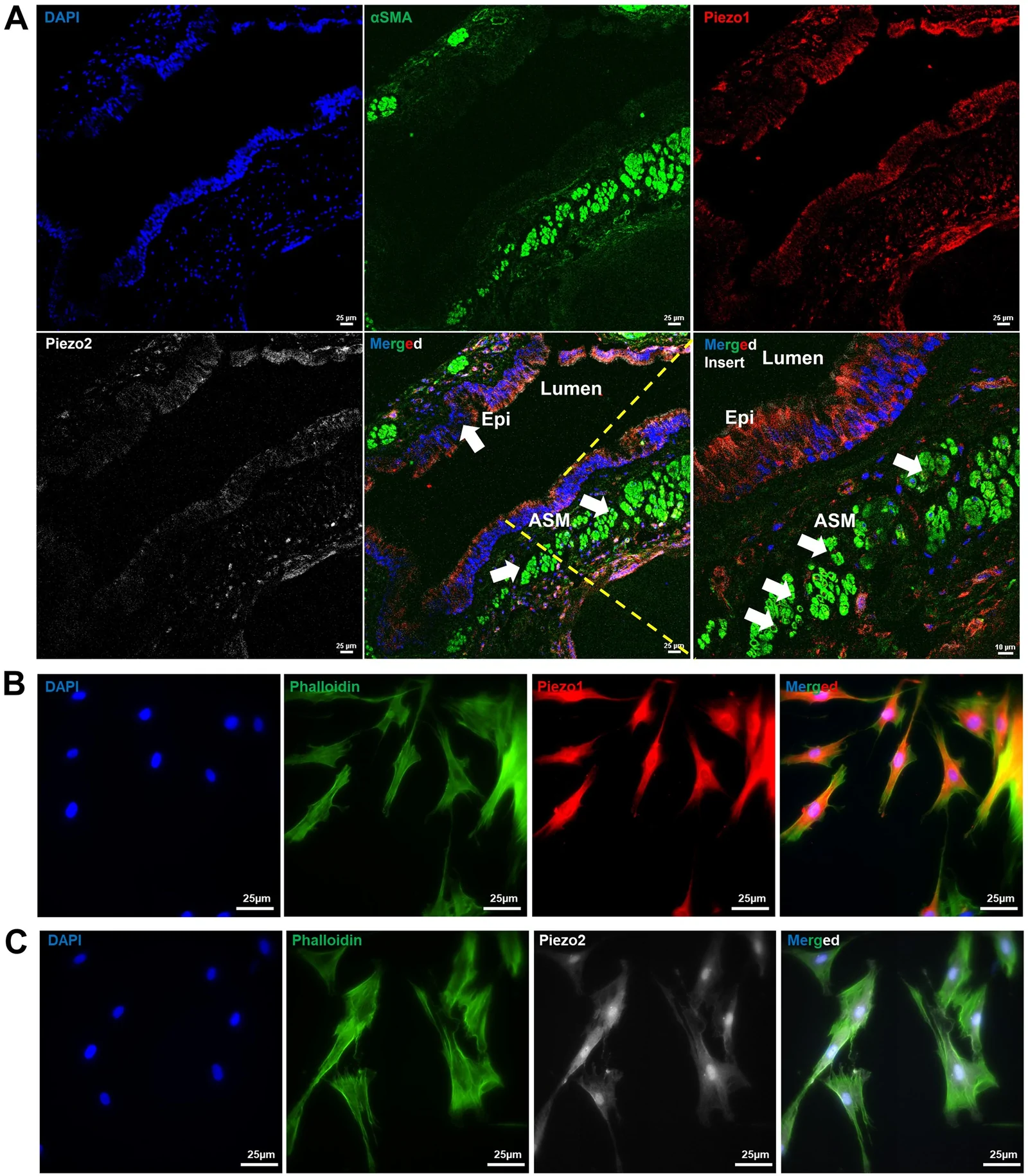
Piezo channel (Pz1 and Pz2) expression in human airways and isolated human airway smooth muscle (ASM) cells. (A) Human lung sections were immunostained for Pz1 (AF-555) and Pz2 (AF-647) with α-smooth muscle actin (α-SMA (AF-488-green)) as a reference marker for smooth muscle colocalization, and DAPI to stain the nucleus (AF-408). Z-stack images were taken on a Nikon confocal microscope. Scale bar: 25 μm; insert: 10 μm. Paraformaldehyde-fixed human ASM cells were immunostained with Pz1 (AF-555); (B) Pz2 (AF-647) and (C) actin filaments were stained with phalloidin (AF-488). DAPI (AF-408) was used for nuclear counterstain. Images were taken on a Nikon Eclipse Ti imaging system. Scale bar: 25 μm. Representative images from 5 independent samples.

### Age- and asthma-related differences in Pz1 and Pz2 expression in ASM cells

Baseline protein levels of Pz1 and Pz2 in primary ASM cells from young, non-asthmatics vs asthmatics and elderly non-asthmatics vs AIE individuals indicated significant expression differences, with AIE ASM showing the highest levels of both proteins compared to the other groups (*P* < .05 for AIE vs elderly non-asthmatic for Pz1; *P* < .05 for young asthmatics vs elderly non-asthmatic; *P* < .01 for elderly non-asthmatic vs AIE for Pz2). This trend persisted after external mechanical stretch, which further increased Pz1 (Figure 2A; *P* < .05) and Pz2 (Figure 2B; *P* < .05) expression across all groups, except for young asthmatics, which showed a trend toward an increase. AIE ASM cells exhibited the highest levels of Pz1 and Pz2 expression compared to young non-asthmatics, young asthmatics, or elderly non-asthmatics. We found significant baseline differences between elderly non-asthmatic and AIE ASM that were not stretched (*P* < .05 for Pz1 and *P* < .01 for Pz2). Interestingly, young asthmatics showed significantly higher Pz2 (*P* < .05) compared to elderly non-asthmatics. Under mechanically stretched conditions, a significant increase in Pz1 expression was observed between young asthmatics vs AIE ASM cells (*P* < .05) and to a greater extent between elderly non-asthmatics and AIE ASM cells (*P* < .001).

**Figure 2:**
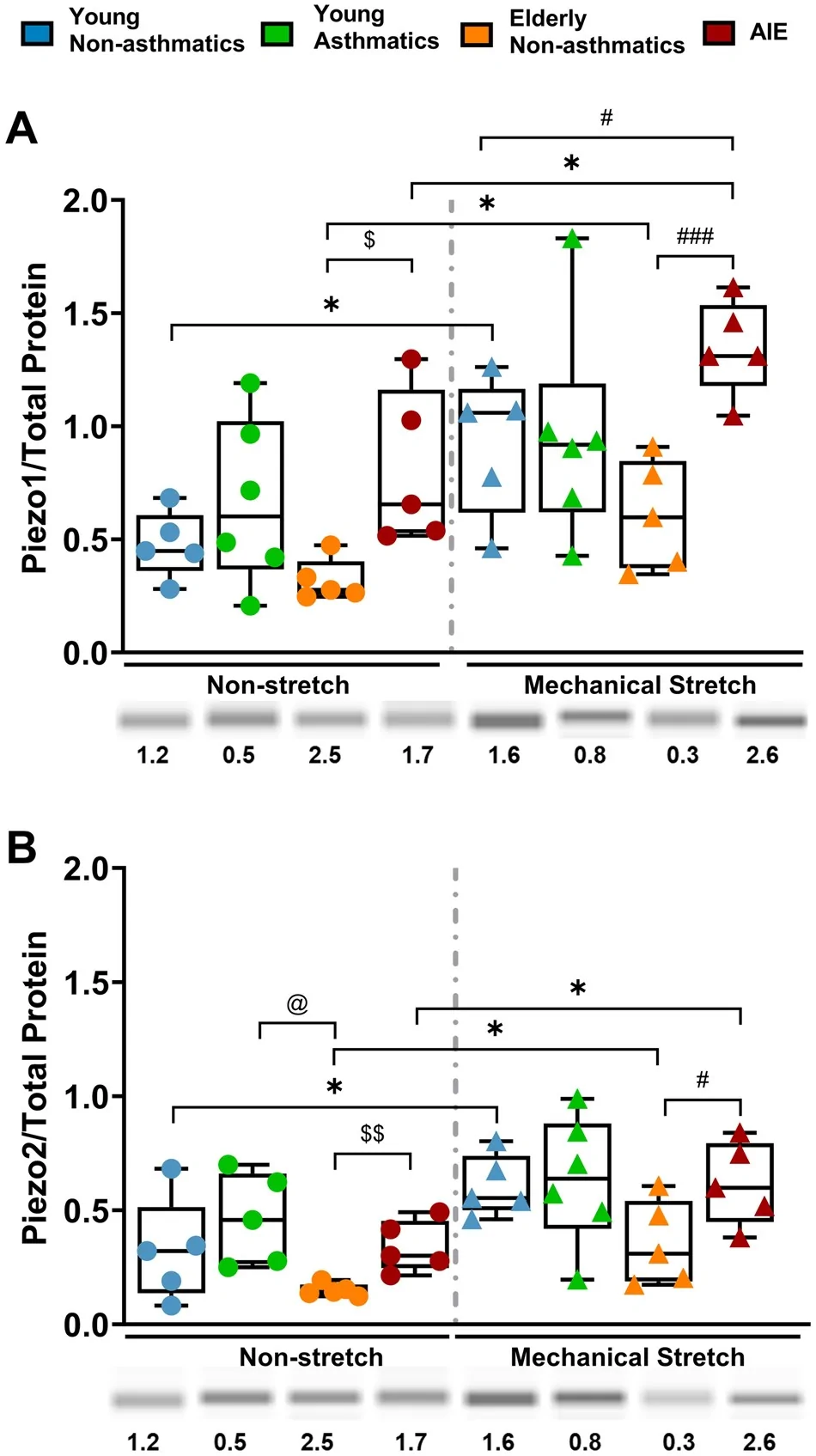
Effect of age, asthma, and mechanical stretch on Pz1 and Pz2 expression in human ASM cells. Capillary electrophoresis-based protein analysis (ProteinSimple JESS) in young vs elderly, non-asthmatics and asthmatics, and AIE ASM showed lower baseline Pz1 (A) and Pz2 (B) expression in ASM cells from elderly patients compared to those from younger non-asthmatic or asthmatic individuals. Pz1 and Pz2 expressions were higher in ASM from elderly individuals with asthma compared to those from younger non-asthmatics or asthmatics and elderly individuals without asthma. *P < .05 vs respective non-stretch counterpart; ^@^*P* < .05 vs non-stretch young asthmatic ASM; ^$^*P* < .05, ^$$^*P* < .01 vs non-stretch elderly ASM; ^#^*P* < .05, ^###^*P* < .001 vs mechanically stretched elderly ASM. Representative electropherograms are shown for Pz1 and Pz2, respectively. *N* = 5-6 patients per group, mean ± SEM for box plots.

### Pz effects on ECM deposition in aging and asthma

In Cell Western analysis of native ECM proteins in human ASM cells demonstrated that activation of Pz1 with Yoda1 led to a significant increase in deposition of collagen I, collagen III, and fibronectin compared to vehicle-treated controls. We observed a trend toward greater collagen I deposition with Yoda1 exposure with age (Figure 3A), an effect that showed a trend toward inhibition by GsMTx4 (Figure 3A) across the groups. In contrast, an increase in collagen I deposition induced by Yoda1 was more substantial in elderly ASM (both non-asthmatic and AIE) cells. Collagen III deposition was generally minimal across all age groups: however, GsMTx4 significantly reduced collagen III deposition in young ASM cells (Figure 3B; *P* < .05). Yoda1 consistently promoted fibronectin deposition in all groups (Figure 3C; *P* < .001 for young and elderly ASM; *P* < .05 for young asthmatic ASM and *P* < .01 for AIE ASM). Such upregulation was effectively blocked by GsMTx4 treatment (Figure 3C; *P* < .05 for all and *P* < .01 for elderly ASM alone). ECM protein levels were normalized to cell numbers determined by bright-field cell count for each condition.

**Figure 3:**
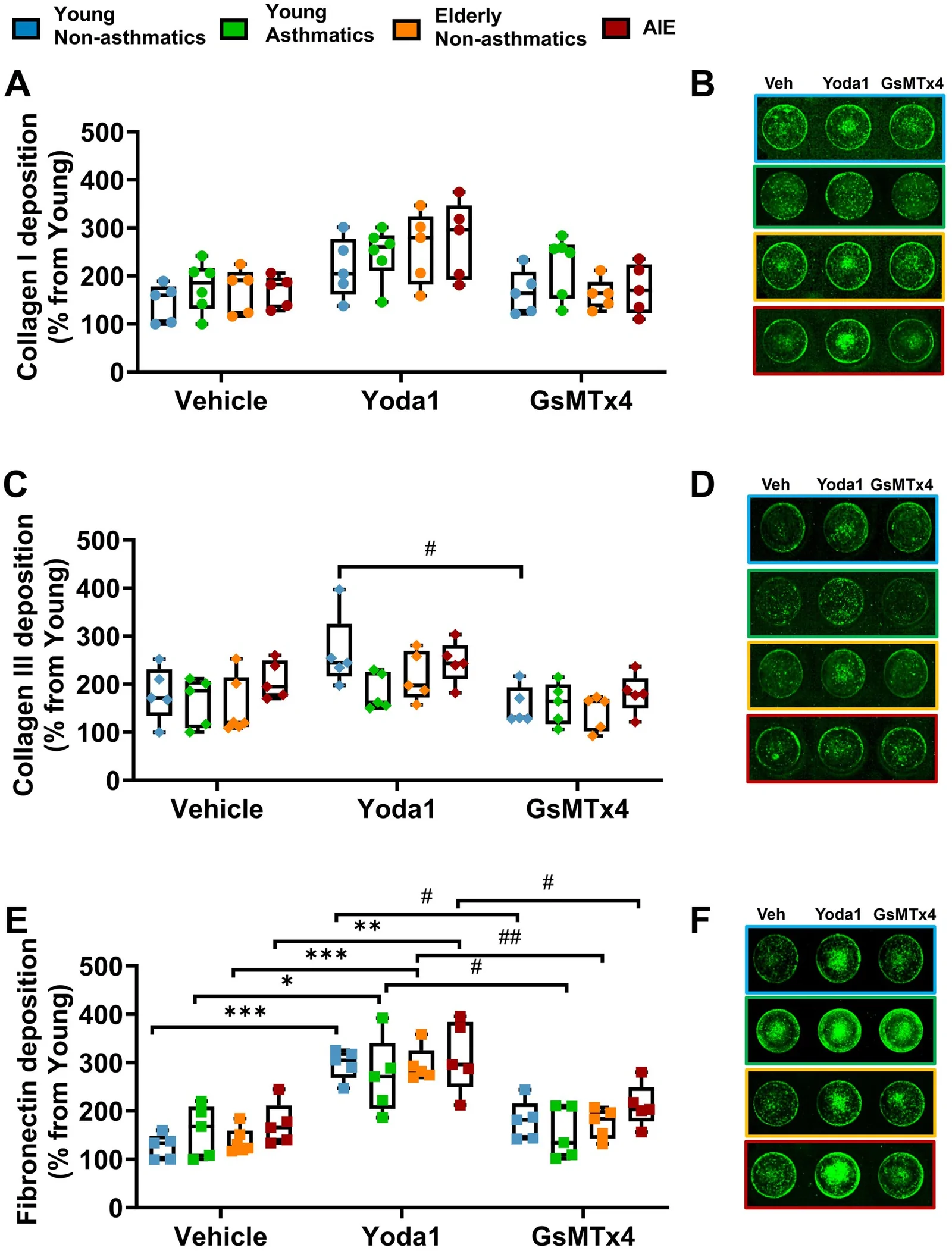
Effect of Pz1 modulation on ECM deposition by human ASM. Deposition of major ECM proteins collagen I (A and B), collagen III (C and D), and fibronectin (E and F) in young vs elderly, non-asthmatics and asthmatics, and AIE ASM were evaluated by In-Cell Western analysis (semiquantitative immunofluorescence of deposited protein, representative examples shown in adjoining panels). Cells were treated with vehicle, Pz1 activator Yoda1 (10 µm), or Pz inhibitor GsMTx4 (1 µm). Yoda1 increased collagen I and III, while GsMTx4 inhibited it, significantly only for collagen III in young ASM. Yoda1 promoted fibronectin deposition in all groups, while GsMTx4 significantly attenuated this increase in fibronectin deposition in ASM from both young and elderly non-asthmatics and asthmatics. *P < .05, **P < .01, ***P < .001 vs respective vehicle; ^#^*P* < .05, ^##^*P* < .01 vs respective Yoda1-treated ASM. *N* = 5 patients per group, mean ± SEM for box plots.

### Pz expression under mechanically stretched conditions in aging and asthma

To explore the mechanosensitivity of Pz channel expression, we subjected human ASM cells from all groups to non-stretch vs mechanical stretch conditions. Mechanical stretch significantly increased Pz1 and Pz2 expressions at baseline for young non-asthmatic (Figure 4A and B; *P* < .05), elderly non-asthmatic (Figure 4E and F; *P* < .05) and AIE (Figure 4G and H; *P* < .05). We did observe a trend toward an increase for the young asthmatic (Figure 4C and D) group; however, this was not statistically significant. Stretch induced increase in Pz expression was further enhanced upon exposure to Yoda1 for young asthmatic and AIE ASM cells (Figure 4C, D, G, and H; *P* < .05 for young asthmatic Pz1 and for both AIE Pz1 and Pz2), and under non-stretch conditions for Pz2 expression for AIE ASM cells alone (Figure 4G; *P* < .05). Notably, mechanically stretched ASM cells exposed to Yoda1 exhibited significantly higher Pz1 and Pz2 expressions compared to non-stretched Yoda1-treated ASM cells (Figure 4B; *P* < .05 for Pz2 in young non-asthmatic and asthmatics and for both-Pz1 and Pz2 in AIE ASM cells), suggesting a greater impact of Pz1 and Pz2 channel activity in AIE ASM cells.

**Figure 4:**
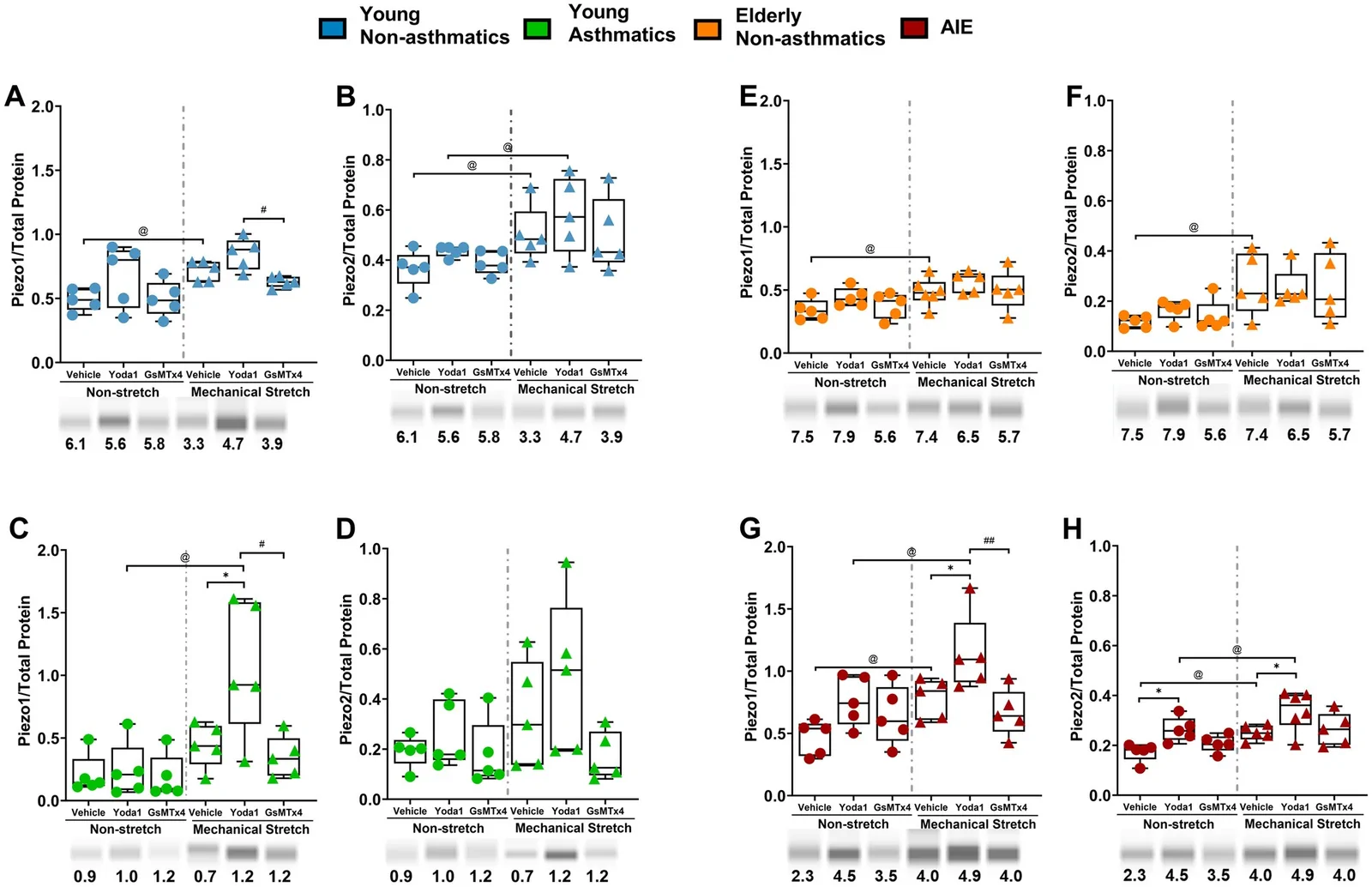
Effect of Pz modulation and mechanical stretch on Pz expression in human ASM. To determine the mechanosensitivity of Pz channel expression, ASM cells from young non-asthmatics (A and B), younger asthmatics (C and D), elderly (E and F), and AIE (G and H) were treated with Yoda1 (10 µm) or GsMTx4 (1 µm) under conditions of no stretch vs mechanical stretch. Mechanical stretch increased Pz1 and Pz2 expression at baseline for all groups, further enhanced by Yoda1 when stretched for young non-asthmatics and asthmatics, more so in AIE ASM. Pz expression was increased at baseline for non-stretch in AIE, suggesting a greater impact of Pz1 and Pz2 channel activity in AIE ASM. *P < .05 vs respective vehicle; ^#^*P* < .05, ^##^*P* < .01 vs respective Yoda1-treated ASM; ^@^*P* < .05 vs non-stretch counterpart. Representative electropherograms are shown underneath the graphs. *N* = 5-6 patients per group, mean ± SEM for box plots.

### Expression of Wnt family proteins in aging and asthma

We next examined whether Pz channel modulation influenced the Wnt signaling pathway in ASM cells from young vs elderly asthmatics vs non-asthmatics. ASM cells were treated with Yoda1 or GsMTx4, with or without mechanical stretch (as above). Mechanical stretch enhanced Wnt3a expression at baseline levels for vehicle alone (Figure 5A, E, and G; *P* < .05), which was further enhanced, particularly in cells exposed to Yoda1 (*P* < .05 for all, except for the young non-stretched group and all of the elderly non-asthmatic ASM cells). Similarly, GsMTx4 treatment was able to abrogate Yoda1-induced Wnt3a expression in mechanically stretched young, non-asthmatic, and elderly ASM cells (Figure 5A, C, and E; *P* < .05), albeit not significantly in AIE ASM cells. Wnt5ab levels were also observed to be significantly higher at baseline vehicle values for AIE ASM cells (Figure 5H; *P* < .05). Yoda1 exposure upregulated Wnt5ab expression in elderly non-asthmatic and AIE ASM only (Figure 5F and H; *P* < .05). Furthermore, GsMTx4 attenuated Wnt5ab expression albeit only in mechanically stretched elderly non-asthmatic ASM (Figure 5F; *P* < .05). At baseline, β-catenin expression was significantly higher in mechanically stretched young, elderly and AIE ASM (Figure 6A, C, and E; *P* < .05). β-catenin expression was markedly increased by Yoda1 treatment (Figure 6A; *P* < .05 for young and Figure 6C and G; *P* < .01 for young asthmatic and AIE, except in elderly non-asthmatic ASM cells), and particularly in asthmatics, indicating Wnt/β-catenin pathway activation under mechanical stretch is potentially exaggerated under asthmatic phenotypes. Here, Yoda1 effect was significantly higher especially in mechanically stretched young asthmatic and AIE ASM (Figure 6C and G; *P* < .01). GsMTx4 abrogated this Yoda1-induced β-catenin expression significantly in mechanically stretched young (Figure 6A; *P* < .05), and to a much greater extent in AIE ASM cells (Figure 6E; *P* < .01). Interestingly, we observed β-catenin expression was attenuated under non-stretched conditions for cells exposed to GsMTx4, but not in mechanically stretched conditions. Although analysis of total GSK3β levels did not reveal conclusive differences (Figure 6B, D, F, and H), we attribute this to the use of total GSK3β instead of active or phosphorylated GSK3β. Future studies will investigate the active or phosphorylated forms of GSK3β in this pathway. Interestingly, the activation of canonical and non-canonical Wnt ligands, Wnt3a and Wnt5ab, along with β-catenin, regardless of mechanical stretch, suggests a role for Pz channels in regulating downstream Wnt signaling.

**Figure 5:**
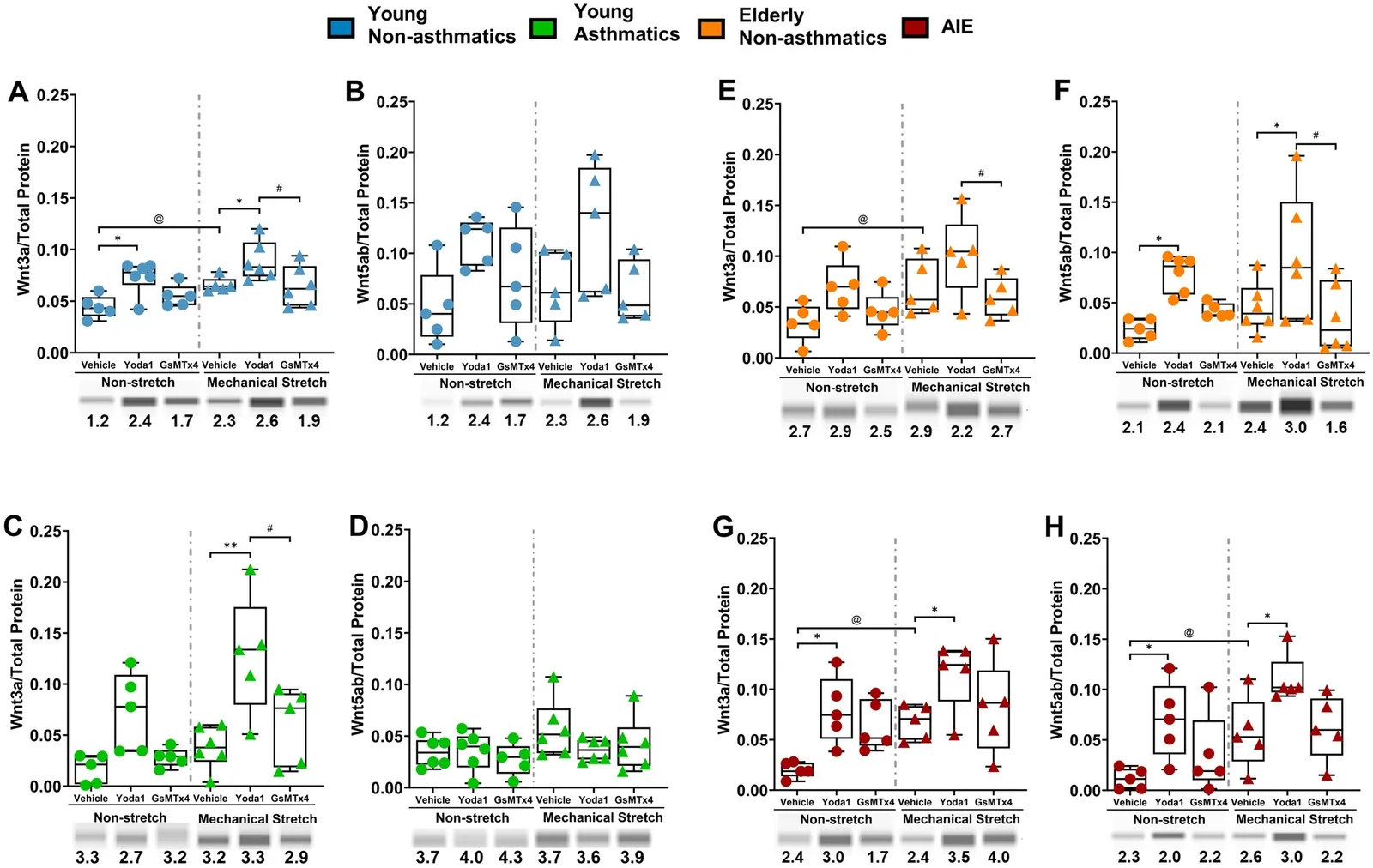
Effect of Pz1 activation on Wnt proteins in human ASM. Wnt3a and Wnt5a expression was determined in ASM cells from young non-asthmatics (A and B), asthmatics (C and D), elderly non-asthmatics (E and F), and AIE (G and H) individuals following exposure to Yoda1 (10 µm) or GsMTx4 (1 µm) under conditions of no stretch or mechanical stretch. Mechanical stretch enhanced Wnt3a expression at baseline, further enhanced by Yoda1, particularly in young asthmatic and AIE ASM. GsMTx4 abrogated Yoda1-induced Wnt3a expression in stretched young non-asthmatics and asthmatics, and elderly non-asthmatic ASM. Wnt5ab levels were also higher at baseline for AIE and Yoda1 upregulated Wnt5ab in elderly and AIE ASM *P < .05, **P < .01 vs respective vehicle, ^#^*P* < .05 vs respective Yoda1-treated ASM; ^@^*P* < .05 vs non-stretch counterpart. Representative electropherograms are shown underneath the graphs. *N* = 5-6 patients per group, mean ± SEM for box plots.

**Figure 6:**
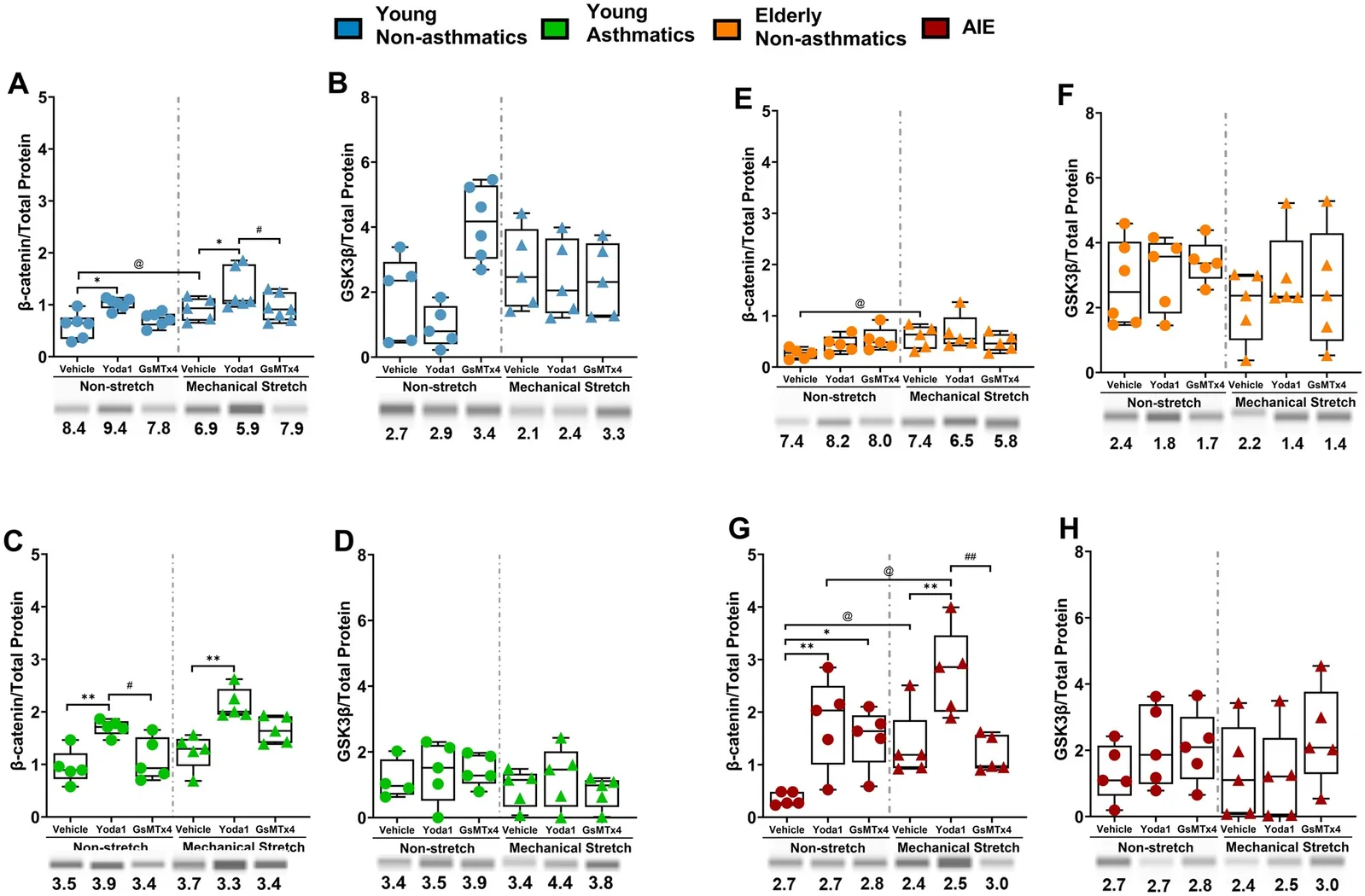
Effect of Pz1 activation on β-catenin and GSK3β in human ASM. Canonical Wnt signaling elements β-catenin and GSK3β expression were determined in ASM cells from young non-asthmatics (A and B), asthmatics (C and D), elderly non-asthmatics (E and F), and AIE (G and H) following exposure to Yoda1 (10 µm) or GsMTx4 (1 µm), under conditions of no stretch and mechanical stretch. At baseline, β-catenin expression was higher in mechanically stretched young non-asthmatics, elderly, and AIE ASM, albeit not significantly for younger asthmatics, and was markedly increased by Yoda1, particularly in young asthmatics and AIE ASM. The Yoda1 effect was higher, especially in mechanically stretched young asthmatic and AIE ASM: effects abrogated by GsMTx4. Analysis of total GSK3β levels did not reveal conclusive differences. *P < .05, **P < .01 vs respective vehicle, ^#^*P* < .05, ^##^*P* < .01 vs respective Yoda1-treated ASM; ^@^*P* < .05 vs non-stretch counterpart. Representative electropherograms are shown underneath the graphs. *N* = 5-7 patients per group, mean ± SEM for box plots.

### Effect of Pz1 on ASM cell proliferation in aging and asthma

Brightfield and Cyquant assays were performed on ASM cells from all groups to evaluate the impact of Pz1 and Pz2 on cell ­proliferation (Figure 7A). To dissect the involvement of Pz1, we treated ASM cells with its activator Yoda1 and inhibitor GsMTx4. Our baseline measurements indicated a non-significant trend toward increased proliferation with age (regardless of asthma) across the groups in the order of young non-asthmatic < young asthmatic < elderly non-asthmatic < AIE. Exposure to Yoda1 enhanced this trend, although the increase was not statistically significant. Conversely, the Pz1 inhibitor GsMTx4 tended to diminish the increase in cell proliferation, resulting in cell counts similar to the control group, although these changes were also not statistically significant.

**Figure 7:**
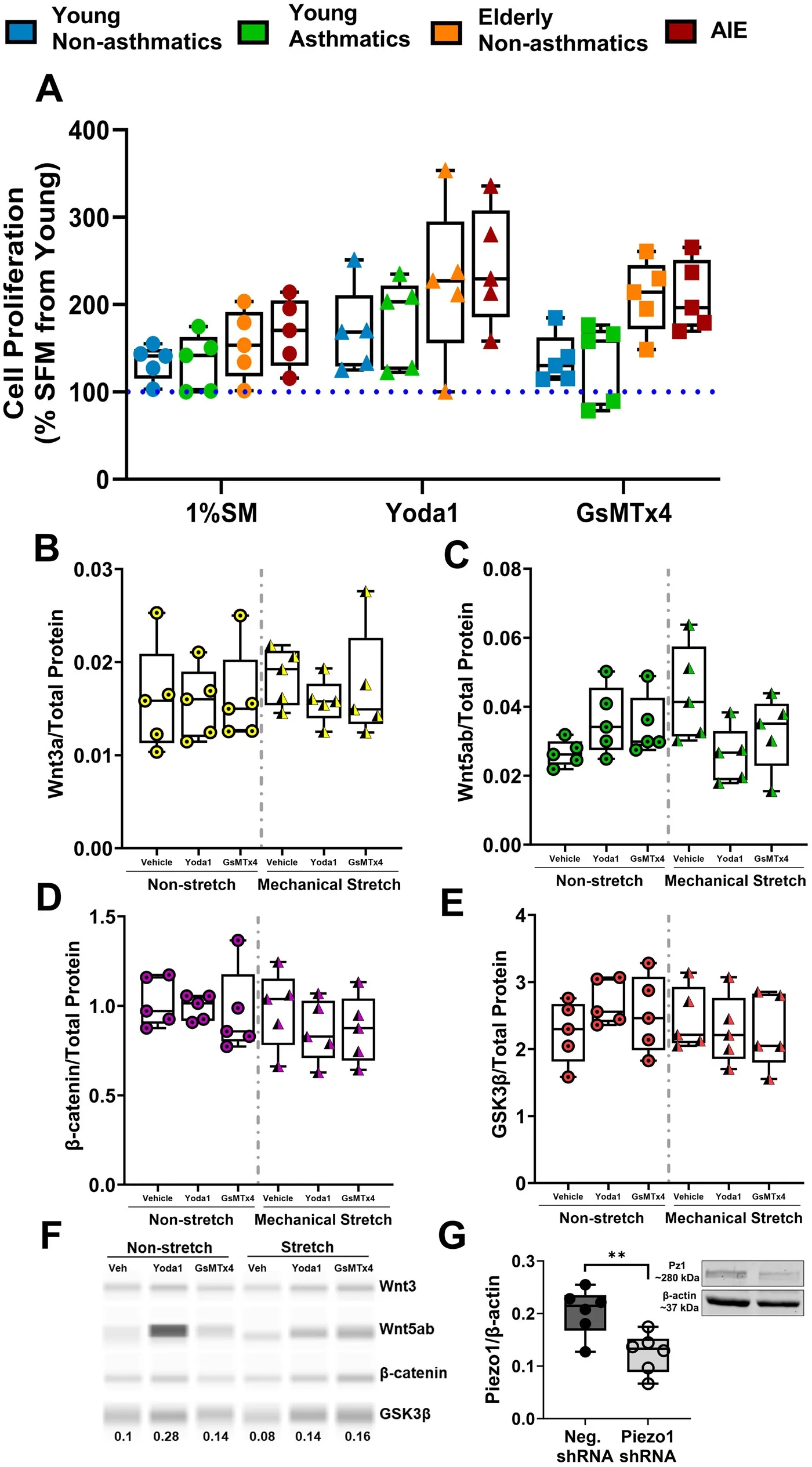
Effect of Pz modulation on ASM proliferation and Wnt signaling. The effect of Pz1 agonist (Yoda1) and antagonist (GsMTx4) on ASM cell proliferation was evaluated using the CyQuant assay in all 4 groups (A). Baseline cell proliferation for young non-asthmatic ASM is indicated by the blue dotted line and used for comparison. Cells were treated with vehicle, Yoda1 (10 µm), and GsMTx4 (1 µm). No consistent pattern of drug, age, disease, or Pz modulation was noted on ASM cell proliferation. Expression of canonical Wnt (Wnt3a) (B), and non-canonical Wnt5ab (C) β-catenin (D) and GSK3β (E) elements was determined in cells where Pz1 channel expression was suppressed by shRNA. ASM cells failed to show the expected increase in these elements, even when exposed to mechanical stretch (representative electropherograms are shown in (F). Cells were verified for knockdown efficiency by using standard Western blot analysis (G) **P < .01 vs negative shRNA. *N* = 5-6 patients per group, mean ± SEM for box plots.

### Effect of Pz1 on Wnt signaling

To directly assess the influence of Pz1 on Wnt signaling, we knocked down Pz1 expression in ASM cells using shRNA-mediated lentiviral transduction (Figure 7G; *P* < .01). Suppression of Pz1 resulted in a decrease in expression of Wnt signaling molecules both under basal conditions and following Yoda1 stimulation. We did not observe significant changes in baseline levels of Wnt3a, Wnt5ab, β-catenin, or total GSK3β in the presence of Pz1 knockdown ASM cells (Figure 7B-F). This suggests that Pz1 contributes positively to the maintenance of Wnt signaling in adult ASM cells.

## Discussion

Our findings provide insights into the contribution of mechanosensitive channels in airway smooth muscle (ASM), particularly Pz1 and Pz2, in airway remodeling with aging and asthma. Using human ASM samples from patients across age groups with/without mild-moderate asthma, we demonstrate the functional implications of Pz channels for cell proliferation, ECM remodeling, and Wnt signaling. We observe expression of Pz1 and Pz2 in human ASM cells and tissues across the age spectrum, with increased expression of both Pz1 and Pz2 in AIE, suggesting a disease-related upregulation. Such upregulation may reflect inflammation in AIE or enhanced signaling resulting from the greater stiffness of aging airways. While we demonstrate an asthma-related (rather than a chronological aging) upregulation in Pz expression, we want to underline that normal aging does not eliminate Pz1-mediated effects; rather, it reduces the magnitude of signaling activation relative to the exaggerated response characteristic of aging asthmatic ASM. Studies on Pz1 activation with Yoda1 vs inhibition of Pz channels with GsMTx4 showed differential effects on cell proliferation, suggesting a modulatory but not critical role of Pz1 in proliferative responses under basal conditions. More compellingly, Pz1 activation significantly enhanced the deposition of ECM proteins across all age cohorts. This effect was present in cells from younger asthmatics but more pronounced in elderly individuals and particularly those with AIE, reinforcing the proposed role of Pz1 in mediating airway remodeling processes in aged asthmatic ASM. A point to note here is that despite lower Pz1 expression, we observed a greater percentage of Yoda1-induced collagen I deposition in elderly non-asthmatic ASM, implying that expression does not mirror functional responsiveness to pharmacological activation. Conversely, pharmacological inhibition of Pz1 demonstrably reduced collagen I and fibronectin deposition, with a particularly potent effect in AIE cells. These findings strongly suggest a role for Pz1 in regulating ECM remodeling, a progressive process in chronic airway diseases like asthma, and a contributor to airway stiffening in aging. Interestingly, these trends became more pronounced under mechanical stretch, highlighting the importance of biomechanical context in Pz1-mediated signaling.

With an increasing elderly population, understanding the impact of normal aging vs aging-associated lung diseases becomes important. In this regard, there has been a focus on the concept of “senile emphysema” with aging.^35^ However, bronchial airways also show increased thickness and fibrosis, suggesting that non-alveolar remodeling is also relevant in normal aging and AIE.^36^ Here, such changes may reflect greater cellular mass and/or increased fibrosis, with limited data suggesting the latter, again highlighting the importance of airway remodeling. However, it is understandably difficult to separate the intrinsic impact of aging itself from the impact of lifelong environmental or lifestyle influences (eg, pollution, smoking, obesity) on airway structure and function that may contribute to age-associated changes or exacerbate AIE.^8^^,^^37^ Furthermore, there is increasing recognition that aging is associated with low-grade inflammation, with factors such as IL-6 playing a role.^38^^,^^39^ Such inflammatory aspects may be exacerbated in AIE, contributing to asthma-related remodeling. Regardless of what factors may be involved, increased airway narrowing with greater wall thickness has been shown in AIE which is consistent with the more severe, less reversible airflow limitations in AIE patients.^9^^,^^40^ Thus, whether AIE is a persistent form of the disease from earlier in life or a new disease later in life, mechanistically, there is likely an introduction or enhancement of factors with aging that were less impactful earlier in life. One factor could indeed be age-related remodeling with/without the mechanical effect of altered airway tethering from senile emphysema, which would overall change the mechanical environment in which cells of the bronchial airway operate.^41^ With increased stiffness, potentially exacerbated by inflammation in AIE, mechanobiological aspects may take on a greater role. Our present study builds on this idea by demonstrating the role of mechanosensitive Pz channels in ASM that contribute to ECM production, particularly in aging and AIE. The focus is on understanding whether mechanobiology and mechanosensitive pathways contribute, perhaps differentially, to the remodeling of aging and AIE. Here, we acknowledge that our study design and the limited sample numbers do not allow for exploring the potential contribution of age per se beyond the grouping of young vs elderly, the confounding effects of environmental exposures, or even inflammation. However, the fact that we do observe an effect of mechanosensitive channels within and across ages, and in AIE, suggests that mechanobiology is a contributory and differentiating mechanism in remodeling in the aging, asthmatic airways.

The relevance of mechanobiology in aging airways has not been well explored. Certainly, the impact of mechanical forces is highly relevant at the other extreme of age, that is, perinatal ages, when continuous positive airway pressure (CPAP) is increasingly used in the neonatal intensive care unit to treat preterm infants with respiratory issues.^42^^,^^43^ While CPAP is beneficial for perinatal outcomes, the longer-term impact of CPAP, particularly on the more compliant developing airways resulting in greater distention when exposed to externally imposed strain, is still under investigation.^44^^,^^45^ Excessive stretch has damaging effects such as inflammation, alveolar simplification, wheezing, and asthma.^46^^,^^47^ Indeed, we have previously found that neonatal mice exposed to CPAP show increased sustained airway reactivity as well as bronchial airway thickening.^48^ In developing human ASM, we have also shown the role of Pz channels in this context, where Pz channels are expressed, and mechanical stretch induces [Ca^2+^]_*i*_ responses that are inhibited by Pz modulation. However, in adults, the mechanical forces of breathing are better transmitted to the airways by tethering parenchyma. Conversely, with larger lungs, the extent of stretch experienced by individual airways and ASM cells in normal breathing is harder to determine. Static stretch becomes relevant in the use of CPAP in sleep apnea, for example, but the impact of such stretch or the role of Pz channels has not been explored. However, with aging, senile emphysema of aging appears balanced by inherent airway stiffening. Yet, airway hyperresponsiveness of AIE imposes stretch and compression on airway cells, overall making the impact of stretch on aging/AIE airway unclear. Our results are the first to demonstrate that stretch does have an impact on aging and particularly ASM cells, which leads to substantially greater ECM production.

ASM cells are critical players in multiple aspects of airway structure and function, as also demonstrated in the context of responsiveness to mechanical stretch and the contribution of Pz channels. We previously reported that aging increases extracellular deposition of collagen III and fibronectin and decreases expression of ECM-modifying proteins MMP2 and MMP9 in human ASM cells.^14^ Analyzing human lung tissues, we found that aging increases collagen and fibronectin expression in bronchial airways and within the ASM layer; aging increases ASM mass area within bronchial airways.^49^ Moreover, these remodeling parameters are further upregulated in elderly people with asthma. Separately, in adult fibroblasts, we found that stretch, via Pz1 channels, modulates the cellular microdomain involving STIM1/Orai1 and intracellular Ca^2+^ with longer-term effects on fibroblast to myofibroblast transmission. Thus, the overall impact of mechanobiology in adult and aging airways and the role of Pz appears important.

We observed a synergistic effect between mechanical stretch and Pz1 activation in modulating components of the Wnt signaling pathway in ASM. Specifically, activation of Pz1 by Yoda1 enhanced the expression of both canonical (Wnt3a, β-catenin) and non-canonical (Wnt5ab) Wnt pathway elements, with this effect being particularly pronounced under conditions of stretch. This finding suggests a multifaceted regulatory role for Pz1, influencing both β-catenin-dependent and β-catenin-independent Wnt signaling cascades. Genetic knockdown of Pz1 using shRNA significantly abrogated the expression of these key Wnt signaling molecules, positioning Pz1 as an upstream regulator of Wnt pathway activation in ASM. These data align with previous investigations highlighting the mechanosensitive nature of Wnt/β-catenin signaling in other tissues, such as vascular and epithelial contexts.^50^ Collectively, our findings support Pz1 as a mechanosensor that transduces mechanical stimuli into intracellular signals that activate core remodeling pathways, including the Wnt pathway, within ASM: a chronic effect that goes beyond the recognized role of Pz1 as a nonspecific cation channel with acute effects on calcium and contractility, for example.

While this study presents evidence supporting the involvement of Pz1 in ASM biology and in aging and AIE, several limitations must be acknowledged. First, while Yoda1 and GsMTx4 are widely used pharmacological tools, off-target effects cannot be entirely ruled out. Furthermore, differentiating between Pz1 and Pz2 is less clear, given the lack of specific modulators for these isoforms. For this reason, we have utilized a loss-of-function approach where we have specifically targeted the Pz1 gene to rule out any off-target effects downstream. Future studies will explore other pharmacological activators of Pz1 such as the newly identified Yaddle1.^51^ Second, the observed trends toward changes in cellular proliferation, despite lacking statistical significance, suggest that proliferation may be influenced by other converging pathways. Separately, while we recognize the critical importance of ASM in airway contractility and hyperresponsiveness in the context of asthma, where Pz channel-mediated Ca^2+^ influx and downstream effects might be important, the current study focused on the understudied chronic effects of remodeling. This does not rule out a role for Pz channel-mediated Ca^2+^ in remodeling per se, but such links remain to be established across the age spectrum and in the context of AIE. Furthermore, while the expression of Pz2 was assessed, its functional contribution was not experimentally interrogated in this study and is being investigated as a future study.

In conclusion, our findings establish Pz1 as a mechanosensitive regulator of both ECM deposition and the downstream Wnt signaling pathway within human ASM, particularly in the context of AIE. This research lays the groundwork for future investigations into targeting mechanosensitive Pz channels, especially Pz1, as a therapeutic strategy to ameliorate airway remodeling in asthma, particularly in AIE.

## Supplementary Material

aanag058_Supplementary_Data
