# Supplementary material for: Piezo1 mediates stretch-induced Wnt/β-catenin signaling and extracellular matrix remodeling in aging asthmatic airway smooth muscle
Source: Am J Respir Cell Mol Biol. 2026 Mar 25;74(10):1376–88. doi: 10.1093/ajrcmb/aanag058 (PMC13624502; doi:10.1093/ajrcmb/aanag058)
Supplement: Supplementary file 1 [file aanag058_supplementary_data.zip › REV2_Supplementary Details_to upload.docx]

**Piezo1 Mediates Stretch-Induced Wnt/β-Catenin Signaling and ECM Remodeling in Aging Asthmatic Airway Smooth Muscle**

**Supplementary Details:**

**Materials and Methods**

**Human ASM Cells**

Human ASM cells were isolated from lung specimens incidental to thoracic surgeries (primarily lobectomies, occasionally pneumonectomies) for focal, noninfectious indications at St. Marys Hospital, Mayo Clinic. Samples were obtained from regions distal from tumor margins. Clinical histories and diagnostic reports were used to classify patients with asthma (only mild-moderate included in this study). Current smokers, patients with other lung diseases (e.g. COPD, fibrosis) or significant CV disease were excluded.

ASM cells were enzymatically dissociated from 3^rd^-5^th^ level bronchi and cultured in Dulbecco’s modified Eagle medium (DMEM)-F12 supplemented with 10% fetal bovine serum (FBS) and 1% antibiotic-antimycotic. Prior to experimentation, cells <passage 5 of subculture were growth-arrested in 1% serum for 24 hours and maintained at 37°C with 5% CO2. All cells were validated and underwent QC prior to use, with phenotype validated by complementary approaches including presence of smooth muscle specific markers (caldesmon, α-smooth muscle actin, smooth muscle MHC) or rapid calcium responses to bronchoconstrictor agonists. Mycoplasma contamination was monitored by PCR on an intermittent basis.

**Chemicals, drugs, inhibitors and antibodies**

Primary antibodies are detailed in the supplement. for Pz1 (NBP1-78537) and Pz2 (NBP1-78624) were obtained from Novus Biologicals (CO, USA). Collagen I (ab34710), collagen III (ab7778), fibronectin (ab2413), Wnt3 (ab81614), β-catenin (ab16051) and GSK3β (ab131356) were purchased from Abcam (Cambridge, UK). Wnt5ab (2530S) and β-actin (4967) were obtained from Cell Signaling Biotechnology (MA, USA).

**Immunofluorescence**

Immunofluorescence was performed 6 μm thick human lung sections. Sections were baked (56°C for 2 hours) and then deparaffinized using xylene and ethanol. Antigen retrieval was achieved by steaming the sections in sodium citrate buffer (pH 6.0), followed by rehydration in Millipore water. Permeabilization was carried out using 0.1% Triton X-100 in 1XPBS, and non-specific binding was blocked with 10% donkey serum. The sections were then incubated with primary antibodies against Pz1, Pz2, and α-SMA. For visualization, Alexa Fluor 488-conjugated secondary antibodies were used to detect α-SMA, while Alexa Fluor 555 and 647-conjugated secondary antibodies were used for Pz1 and Pz2, respectively. Nuclei were counterstained with DAPI-AF408. High-resolution Z-stack images of the stained sections were acquired using a Nikon confocal microscope.

For immunofluorescence in human ASM cells, cells were fixed with 4% paraformaldehyde in 1XPBS (pH 7.2), washed three times with PBS, permeabilized with 0.1% Triton X-100 in PBS, and washed again three times with PBS. After blocking with 10% donkey serum, the cells were incubated overnight at 4°C with either a polyclonal rabbit anti-Pz1 antibody or a polyclonal rabbit anti-Pz2 antibody in separate wells. The primary antibodies were then detected using Alexa Fluor 555 (for Pz1) and Alexa Fluor 647 (for Pz2) secondary antibodies. Phalloidin was used to mark smooth muscle, and nuclei were counterstained with DAPI-AF408. Images were captured using a Nikon Eclipse Ti imaging system.

**Cell proliferation**

***Bright-Field Cell Counting:*** Following 24 hours of the different treatments, the total number of cells in each well of the 96-well plates was determined using a high-contrast, bright-field, direct cell counting method on the Flex Station3 microplate reader (Molecular Devices, CA, USA).

***CyQuant Proliferation Assay:*** For all experiments, 24 hours after the designated treatments, the cells were washed with PBS and then incubated with the CyQuant dye for 1 hour at room temperature. The fluorescence resulting from dye binding to DNA, which indicates the number of cells, was measured using the Flex Station3 microplate reader. Treatments were conducted in media containing 1% serum or the corresponding vehicle. The extent of proliferation was normalized to the baseline and is presented as the percentage of proliferation relative to young ASM cells treated with serum free media (SFM) (defined as time zero).

**ECM deposition**

The total number of cells in each well was quantified using high-contrast bright-field direct cell counting on the Flex Station3. Following cell counting, the cells were removed by decellularization with 0.016 N NH4OH for 45-60 minutes, a process visually confirmed by microscopy to ensure only the naturally deposited ECM remained. The plates were then washed three times with PBS, and non-specific protein binding was blocked with Li-Cor Odyssey Blocking Buffer (927-40000) for 1 hour before overnight incubation at 4°C with primary antibodies against collagen I, collagen III, and fibronectin. After washing again, the cells were incubated for 60 minutes with infrared dye-conjugated secondary antibodies. ECM deposition was then measured using a semi-quantitative immunofluorescence In-Cell Western technique with a Li-Cor Odyssey XL System, and densitometry was used for quantification. The expression levels of ECM proteins were normalized to the cell number that was determined by high-contrast bright-field direct cell counting on each plate before the cells were removed.

**Protein Analyses**

***JESS:*** Protein expression levels were assessed using the JESS capillary electrophoresis system (ProteinSimple, CA, USA) according to the manufacturer's instructions. For each analysis, 0.3 µg of cellular protein was loaded onto JESS separation modules, utilizing both 12–230 kDa and 66–440 kDa cartridges. The proteins of interest (Pz1, Pz2, Wnt3, Wnt5ab, β-catenin, and GSK3β) were targeted with specific primary antibodies diluted to an optimized ratio in antibody buffer, followed by horseradish peroxidase (HRP)-conjugated secondary antibodies. Protein expression was normalized to the total proteinx10^5^ loaded. These are represented in bold underneath each band. Digital representations of the resulting electropherograms were generated and subsequently quantified using Compass for Simple Western Software.

***Western Blot:*** For Western Blot analysis, approximately 30 µg of each lysate were separated by 10% SDS-PAGE and then transferred onto 0.22 µm PVDF membranes. Non-specific binding was blocked with 5.0% bovine serum albumin (BSA), and the membranes were incubated overnight at 4°C with the relevant primary antibodies. Following this, the blots were incubated with Li-Cor secondary antibodies. Protein expression detection and densitometry were performed using a Li-Cor Odyssey IR scanning system (NO, USA). The intensity of the protein bands was normalized to the β-actin signal.

**shRNA lentiviral particle transduction**

ASM cells were grown in 6-well plates until they reached approximately 50-60% confluency. Transfection was performed using 20 μL of viral stock containing 1 × 10⁵ infectious units of lentiviral particles for either a control (negative) shRNA or a Pz1 shRNA. When the cells reached 60-70% confluency, the growth medium was switched to fresh 5% serum medium (without antibiotics) containing polybrene (Santa Cruz Biotechnology; sc-134220). The thawed lentiviral particles were added to the cells, and they were incubated overnight. The medium was then replaced with 5% serum medium containing 1% antibiotic-antimycotic (AbAm) but without polybrene, and the cells were incubated for an additional 24 hours to allow for viral transduction. Following this, the medium was changed to 10% serum medium with antibiotics and incubated for 48 hours to promote cell growth. To select for cells that had stably incorporated the shRNA, the medium was replaced with 10% serum medium containing 5 μg/mL puromycin, and the cells were incubated for 48 to 72 hours. The puromycin-containing medium was replenished every 3 to 4 days until several puromycin-resistant colonies were observed. Once these colonies reached 50% confluency, the cells were expanded by transferring them to T-25 or T-75 cell culture flasks. The effectiveness of the shRNA transduction and the knockdown of the target protein were confirmed using Western blot analysis.

**Table1:**

| **Classification** | **Age (years)** | **Asthma status** | **Sex**  **Male(M)/**  **Female(F)** | **Reason of surgery** | **FEV_1_/ FVC** |
| --- | --- | --- | --- | --- | --- |
| **Young non-asthmatics** | 42 | Non-asthmatic | F | Lung Nodule | - |
|  | 33 | Non-asthmatic | M | _- | 84 |
|  | 44 | Non-asthmatic | M | Pulmonary nodule | 84 |
|  | 37 | Non-asthmatic | F | Left lung endobronchial lesion | 93 |
|  | 41 | Non-asthmatic | F | Metastatic sarcoma to the lung | 109 |
|  | 40 | Non-asthmatic | F | transplant | 88.5 |
| **Young asthmatics** | 54 | Mild | F | Adeno | 68 |
|  | 53 | NOS | M | Lung tumor | - |
|  | 56 | NOS | M | - | 68 |
|  | 45 | NOS | M | Lung carcinoma | 69 |
|  | 31 | NOS | M | Lung tumor | 69 |
|  | 54 | Mild | M | Lung nodule | - |
| **Elderly**  **non-asthmatics** | 67 | Non-asthmatic | M | Adeno | - |
|  | 67 | Non-asthmatic | F | Pulmonary nodule | 102 |
|  | 65 | Non-asthmatic | F | Lung nodule | 67 |
|  | 65 | Non-asthmatic | F | Adeno | 103 |
|  | 74 | Non-asthmatic | F | Pulmonary nodule | 71 |
|  | 69 | Non-asthmatic | M | Malignant neoplam of lower lung | 102 |
| **AIE** | 76 | Mild | M | Pulmonary nodule | 68 |
|  | 74 | NOS | F | Lung carcinoma | - |
|  | 77 | NOS | M | Lobectomy | 52 |
|  | 65 | Mild | M | Lung tumour | 68 |
|  | 71 | Mild | M | Lung tumour | 44 |
|  | 79 | NOS | M | Lung carcinoma | - |

Pulmonary function testing demonstrated distinct differences in airflow limitation across groups. For one young and elderly non-asthmatic and two young and AIE, no pulmonary function data was available. The FEV₁/FVC ratio was preserved in young non-asthmatic (88.5 ± 11.78), consistent with normal pulmonary mechanics. In contrast, younger asthmatics exhibited a reduced FEV₁/FVC ratio (68.5 ± 0.57), indicating airflow obstruction with minimal inter-subject variability. Elderly non-asthmatics showed an elevated FEV₁/FVC ratio (102 ± 18.31), suggestive of a restrictive pattern or reduced lung volumes, while AIE individuals demonstrated a markedly decreased FEV₁/FVC ratio (60 ± 12), consistent with moderate-to-severe obstructive physiology. NOS: Not otherwise specified/Asthma severity not specified; FEV_1_/FVC: Forced Expiratory Volume in 1 second/ Forced Vital Capacity.
